# A Case of Rattlesnake Bite Successfully Treated with Permanganate of Potash

**Published:** 1885-08

**Authors:** B. Hall Smith


					﻿A CASE OF RATTLESNAKE BITE SUCCESSFULLY
TREATED WITH THE PERMANGANATE OF
POTASH.
By B. HALL SMITH, M. D.
Doubtless some of the readers of The Journal have seen an
article claiming the permanganate of potash as an efficient rem-
edy in combatting the poison of cobra.
This plan of treatment was first instituted by an English sur-
geon, whose name I have forgotten, he receiving from the Brit-
ish government a large reward for its discovery. My attention
was called to this subject early in the summer of 1882, by an ar-
ticle in the Medical News of Philadelpnia, and I determined to try
this remedy, should the opportunity ever present itself, with
the rattlesnake or moccasin. The following season I treated the
case which I now present to your notice:
While practicing medicine at Graham, Appling county, Ga.,
I was summoned at sundown on the afternoon of the 31st of May,
1883, to attend John Myers, a farmer, who had been bitten by a
large rattlesnake. Being somewhat delayed in responding im-
mediately, I gave the messenger (the victim’s son) a bottle of
pure carbolic acid, instructing him to make all possible haste, cut
deep down into the punctures made by the fangs of the serpent,
and pour in some of the acid. Arming myself with a bottle of
whisky (the procuring of which had caused my delay), and a
bottle of permanganate of potash, I mounted a swift horse, and
soon reached the bedside of the injured man, a distance of six
miles from my office. I found him completely under the influ-
ence of the poison, the symptoms of snake-bite showing them-
selves in a marked degree; exceeding cardiac weakness, dilata-
tion of the pupil, and great prostration. The directions given his
son had been thoroughly carried out, and the wound was well
cauterized. I at once attempted to give him some whisky, but
found his stomach too irritable to retain it, he, unfortunately,
through the advice of some friends, having swallowed a quantity
of tobacco juice, which produced such nausea as to render fu-
tile any effort to administer medicine by the stomach.
I at once resorted to my hypodermic syringe, and gave a num-
ber of injections of whisky, inserting the needle deeply into the
tissues of the deltoid muscle. Immediately following, I began with
the permanganate of potash, dissolving grains in 30 minims
of water, and injecting it into the tissues of the leg and arm; this
I repeated every twenty minutes or half hour, alternating with
whisky and an occasional dose of diluted spirits of ammonia.
I continued these remedies by the hypodermic method for
three hours, when the pain of the sufferer became so great that I
gave him the third of a grain of morphia, which somewhat re-
lieved his restlessness. I continued my efforts, as described, for
two hours more, when Dr. James H. Latimer, of Ilazelhurst,
Ga., came to my assistance, and the result of our consultation as
to prognosis was, that in an hour the case would terminate fatally,
Dr. L. remarking at the time that this was the twelfth case of the
kind he had been called to, and the only one in which he found
the patient alive when he reached him.
Dr. Latimer agreeing with me regarding the treatment, we ex-
erted ourselves for an hour longer, at the end of which time the
patient had become so much worse that we abandoned all hope,
and his relatives and friends assembled around his bed to await
the fatal issue. Indeed, he had every appearance of immediate
dissolution, and it seemed as though every breath would be his
last. At this juncture I asked permission to make one more ef-
fort to save him; my request was reluctantly granted by his fam-
ily. I then gave him a number of hypodermic injections of
whisky, and perhaps one of sulph. ether, and afterwards the
permanganate of potash. In a few minutes his condition was less
alarming, and with renewed hope we urged the same treatment
with such good results that at the end of two hours he was so
much improved that I was permitted to leave him for a short
time. This improvement continued, and by morning I consid-
ered him almost out of danger.
At 8 o’clock, being called away, I left Dr. Latimer in charge,
through the advice of some friends, having swallowed a quantity
of tobacco juice, which produced such nausea as to render fu-
tile any effort to administer medicine by the stomach.
I at once resorted to my hypodermic syringe, and gave a num-
ber of injections of whisky, inserting the needle deeply into the
tissues of the deltoid muscle. Immediately following, I began with
the permanganate of potash, dissolving grains in 30 minims
of water, and injecting it into the tissues of the leg and arm; this
I repeated every twenty minutes or half hour, alternating with
whisky and an occasional dose of diluted spirits of ammonia.
I continued these remedies by the hypodermic method for
three hours, when the pain of the sufferer became so great that I
gave him the third of a grain of morphia, which somewhat re-
lieved his restlessness. I continued my efforts, as described, for
two hours more, when Dr. James H. Latimer, of Ilazelhurst,
Ga., came to my assistance, and the result of our consultation as
to prognosis was, that in an hour the case would terminate fatally,
Dr. L. remarking at the time that this was the twelfth case of the
kind he had been called to, and the only one in which he found
the patient alive when he reached him.
Dr. Latimer agreeing with me regarding the treatment, we ex-
erted ourselves for an hour longer, at the end of which time the
patient had become so much worse that we abandoned all hope,
and his relatives and friends assembled around his bed to await
the fatal issue. Indeed, he had every appearance of immediate
dissolution, and it seemed as though every breath would be his
last. At this juncture I asked permission to make one more ef-
fort to save him; my request was reluctantly granted by his fam-
ily. I then gave him a number of hypodermic injections of
whisky, and perhaps one of sulph. ether, and afterwards the
permanganate of potash. In a few minutes his condition was less
alarming, and with renewed hope we urged the same treatment
with such good results that at the end of two hours he was so
much improved that I was permitted to leave him for a short
time. This improvement continued, and by morning I consid-
ered him almost out of danger.
At 8 o’clock, being called away, I left Dr. Latimer in charge,
and did not return until 5 o’clock in the afternoon. During my
absence the syringe got out of order, and the milk that was given
him was immediately vomited; however, I had another syringe
and gave him grain of morphia with a happy effect; he was
soon able to take nourishment ad libitum.
There was considerable sloughing around the wound, but re-
pair was rapid and his recovery uninterrupted.
				

